# Fibers beyond structure: do they contribute to embolism reversal after drought relief in poplar?

**DOI:** 10.1111/nph.70179

**Published:** 2025-05-01

**Authors:** Niccolò Tricerri, Martina Tomasella, Silvia Cavalletto, Francesco Petruzzellis, Sara Natale, Alan Crivellaro, Rachele Gamba, Alma Piermattei, Lorenzo D'Amico, Giuliana Tromba, Andrea Nardini, Maciej A. Zwieniecki, Francesca Secchi

**Affiliations:** ^1^ Department of Agriculture, Forest and Food Sciences University of Turin Largo Paolo Braccini 2 10095 Grugliasco Italy; ^2^ University School for Advanced Studies IUSS Pavia 27100 Pavia Italy; ^3^ Dipartimento di Scienze della Vita University of Trieste via Giorgieri 10 34127 Trieste Italy; ^4^ Department of Biology University of Padova Via Ugo Bassi 58B 35121 Padova Italy; ^5^ Forest Biometrics Laboratory, Faculty of Forestry “Stefan cel Mare” University of Suceava, Str. Universitatii 13 720229 Suceava Romania; ^6^ Elettra‐Sincrotrone Trieste Area Science Park, Basovizza 34149 Trieste Italy; ^7^ Department of Plant Sciences University of California Davis One Shields Ave 95616 Davis CA USA

**Keywords:** drought, embolism, fibers, microCT, poplar, recovery, vessels

## Abstract

- Short‐term recovery from drought‐induced vessel embolism is an energy‐dependent biological process that requires a water source and solutes, both likely supplied by parenchyma cells. Despite fibers primarily providing structural support, their functional role as a reservoir of unbound water during and after stress remains unclear.
- In this study, *Populus nigra* plants were exposed to two drying regimes (slow and fast developing stress). At the end of the drought treatments and after stress relief, nondestructive structural observations were performed *in vivo* using synchrotron X‐ray microCT.
- Different drought progression rates did not affect the final extent of vessel embolism, but poplars subjected to slower drought development exhibited higher levels of air‐filled fibers. Following stress relief, faster hydraulic recovery was observed in plants exposed to rapid drought, which displayed lower occurrences of water‐depleted fibers.
- We suggest a novel functional role for xylem fibers during drought and recovery. We hypothesize that parenchyma cells can access water stored in completely mature fibers via pits, enhancing their survival during drought. Upon xylem tension relief, this stored water may be mobilized by living cells from fibers to vessels, facilitating the recovery of their transport function.

## Introduction

Xylem is a complex bio‐physical system that provides multiple functions, including water and nutrient transport, structural support, energy and water storage, self‐healing, and protection (Tyree & Zimmermann, 2002). Multiple evolutionary innovations in vascular plants implied increasing structure‐to‐function efficiency, achieved through the separation of these functions into highly specialized tissues and cells. In the case of angiosperms, the separation of water transport from structural support is one of the evolutionary milestones allowing this group to dominate a wide range of climatic conditions, from wet tropical and arctic regions to dry warm and cold deserts (Willis & McElwain, 2002). In particular, the evolution of vessel elements with perforation plates led to the reduction in long‐distance hydraulic resistance, and their increased diameter compared with tracheids allowed for reducing the number of conduits necessary to meet hydraulic demands. However, this reduction in hydraulic redundancy led to the need for protective structures and mechanisms to cope with cavitation risk and the formation/spread of embolism. Connections between vessels, called intervessel pits, are the first line of defense against hydraulic failure, as the air–water interface can be upheld on a homogeneous cellulose membrane that separates adjacent vessels (Schmid & Machado, 1968; O'Brien, 1970; Plavcová & Hacke, 2011; Stroock *et al*., 2014). Typically, the vessel lumen fraction remains low, below 20% of total xylem area (Avila *et al*., 2023), the remaining area being composed of axial and radial living parenchyma cells, as well as mechanically functional (structural) and not‐yet mechanically functional (living) fibers. Dead fibers, which often constitute the largest share of xylem volume (Dória *et al*., 2022), provide structural support. Given that cell walls can make up to 50% of the fiber volume (Jacobsen *et al*., 2005), it is plausible that the remaining lumen volume may provide water reserves to dampen sudden changes in water potential or to favor postdrought recovery from embolism.

When facing drought, plants close stomata to reduce evapotranspiration, thus buffering xylem water potential drop and delaying xylem embolism build‐up, which is the main cause of stem water transport capacity loss (Zeppel *et al*., 2019). Plants can eventually recover from embolism by adopting two nonexclusive physiological strategies: the formation of new functional xylem (a long‐term strategy) and the restoration of the functionality of embolized conduits (a short‐term strategy). A growing body of evidence has shown that, for some species, the latter represents a viable strategy to recover from water stress under low xylem tension (Johnson *et al*., 2012; Brodersen & McElrone, 2013; Zwieniecki *et al*., 2013; Savi *et al*., 2016; Trifilò *et al*., 2017; Klein *et al*., 2018; Secchi *et al*., 2021). Although this physiological process is not fully elucidated, it has been suggested that living parenchyma cells in the immediate proximity of embolized vessels serve as the source of both osmotically active solutes and water needed for the recovery of hydraulically isolated conduits (Secchi *et al.,* 2011, 2017). *In vivo* observations have shown that during recovery vessels fill up with water (Holbrook *et al*., 2001; Scheenen *et al*., 2007) initially derived from water droplets that preferentially form and grow on vessel walls in contact with living parenchyma cells (Brodersen *et al*., 2010). As the volume of living parenchyma cells is relatively small, also considering that these cells need to remain turgid, it is unlikely that they can alone provide the large amounts of water needed to refill empty vessels, prompting questions on the actual sources of the water used for hydraulic recovery. One possible source of water is the phloem, which delivers both water and solutes to the embolized conduits through parenchyma rays, as shown in experiments damaging phloem continuity which lead to decreased capacity for recovery (Bucci *et al*., 2003; Salleo *et al*., 2004; Nardini *et al*., 2011). Another possible source could be the water stored in xylem fibers, which interestingly constitute the largest pool of unbound water in xylem, that may (Suuronen *et al*., 2013; Chu *et al*., 2023) or may not (Knipfer *et al*., 2017, 2019) reduce the risk of embolism occurrence. Detailed observations of the temporal dynamics of water in fibers' lumina during water stress and recovery might help to answer several questions: Do fibers release stored water to provide a temporary buffer alleviating xylem tension to avoid xylem embolism, and/or do they release water only during recovery thus serving as a possible source of water to sustain hydraulic recovery?

Until recently, it was virtually impossible to study the water status of wood *in vivo* at high spatial resolution. However, progress in phase‐contrast micro‐computed tomography (PCμCT) allowed for observations of living stems to quantify and localize vessel embolization (Suuronen *et al*., 2013; Cochard *et al*., 2015; Nardini *et al*., 2017; Nolf *et al*., 2017; Secchi *et al*., 2021). In fact, μCT reconstructions are characterized by an excellent contrast between air‐filled and water‐filled conduits based on the high delta in X‐ray attenuation values between them. More recent technical advances increased the resolution of images to below 1 μm, thus allowing for the localization of fibers in xylem and the determination of their water status. Our objective was to exploit PCμCT technology to observe the temporal and spatial dynamics of vessel and fiber water status in plants exposed to severe water stress imposed over different time intervals, as well as during the recovery from drought.

Previous studies suggested that slow water stress progression can delay the rate of recovery and the success of refilling (Trifilò *et al*., 2017; Morabito *et al*., 2021), and this was attributed to effects on the metabolism of wood nonstructural carbohydrates (NSCs). However, a biophysical explanation for the phenomenon is still lacking. Here, we exposed poplar plants to two types of drying regimes (a slow 4‐wk and a fast 1‐wk drying time) to observe *in vivo* how drought kinetics affect the amount of vessel embolism and the ability to recover after water stress. Experiments were conducted on *Populus nigra* L. saplings, a diffuse‐porous wood species with vessels embedded in a xylem matrix primarily composed of fibers, with simple to minutely bordered pits, and radial parenchyma cells. Our results highlight different embolism recovery potential based on stress progression dynamics and suggest a functional role for fibers in restoring hydraulic connectivity, as water reserves for living tissues during and after drought. In addition, we introduce novel semi‐automated approaches to measure fiber–conduit connections and the positions of embolized vessels and fibers, which can serve as useful tools for quantifying these parameters.

## Materials and Methods

### Plant materials and growth conditions

Cuttings of black poplar (*Populus nigra* L.), provided by the nursery Vivaio Gambarello (Chiusa Pesio CN, Italy), were rooted in 2‐l pots filled with a substrate composed of a sandy‐loam soil : expanded clay : peat mixture (2 : 1 : 1 by weight). The cuttings were grown for 3 months in a controlled environment room at the University of Turin (45.066317, 7.591606) under partially controlled climatic conditions, with an average air temperature of *c*. 20°C and relative humidity of 44%. A total of 36 plants, with an average height of 26.5 ± 5.8 cm at the beginning of the experiment, were used in this study. The plants were daily irrigated to pot capacity until the beginning of the experimental drought treatments. Plants were initially divided into two groups: 23 plants were irrigated every morning to maintain pot capacity weight; 13 plants were slowly water stressed by gradually reducing the daily water supply (restoring the 70% of the water lost from the previous day; SDD, slow‐developed drought). The weight of all the pots was recorded every morning. On day 18, the well‐watered plants were split into two groups of 10 and 13 plants: well‐watered control (CTR, *n* = 10) and plants subjected to rapid water stress by withholding irrigation (FDD, fast‐developed drought, *n* = 13). For both groups of stressed plants (SSD and FDD), the single drought was imposed and maintained until the stem water potential (Ψ_stem_) dropped below −2.0 MPa, a value typically inducing 50% PLC in poplar (Secchi & Zwieniecki, 2012). Plant height was recorded at regular intervals throughout the entire drought experiment (24 d). At the end of the stress treatments, 8 SDD and 8 FDD plants were rewatered in the morning to full pot capacity (as mentioned in the next section) and allowed to recover over a period of 24 h. These plants were denoted as R_SDD (recovered from slow‐developed drought) and R_FDD (recovered from fast‐developed drought).

### Stem water potential measurements

The stem water potential (Ψ_stem_) was periodically measured throughout the duration of the experiment (between 9:00 a.m. and 12:00 p.m.) and before PCμCT imaging, using a Scholander‐type pressure chamber (Model 1505D; PMS Instrument Co. Albany, OR, USA). In brief, leaves were covered with aluminum foil and placed in a humidified plastic bag for at least 20 min before excision. After excision, leaves were allowed to equilibrate for at least 20 min in dark conditions before measurements.

### Phase‐contrast micro‐computed tomography observations

Water‐stressed and well‐irrigated intact poplar saplings were transported to the Elettra Sincrotrone Trieste facility. Here, part of the SDD‐ and FDD‐stressed plants were rewatered to pot capacity (as described earlier). Phase‐contrast micro‐computed tomography observations were performed at the SYRMEP beamline on intact CTR (*n* = 6), SDD (*n* = 5), and FDD (*n* = 4) plants as well as on R_SDD (*n* = 8) and R_FDD (*n* = 4) recovered poplars (24 h after stress relief). To prevent potential tissue damage caused by ionizing radiation (Petruzzellis *et al*., 2018), each stem was exposed to only one scan. Before PCμCT scanning, to avoid water loss and to reduce sample movements during scan rotation, the poplar saplings were quickly wrapped in plastic film and secured to a sample holder (Tomasella *et al*., 2024). The PCμCT analyses were performed in propagation‐based phase‐contrast modality using an Orca Flash 4.0 sCMOS, coupled with a 17‐μm GGG scintillator as a detector. The sample was placed at a distance of 15 cm from the detector. The experiment was conducted in the white beam mode with a 1.0‐mm silica filter, resulting in a mean X‐ray energy of *c*. 22 keV. The exposure time was set at 100 ms, and the scans were performed on the stem at *c*. 25 cm from the apical meristem. In total, 2.048 slices per sample with a pixel resolution of 1 μm were reconstructed using the software SYRMEP TOMO PROJECT (Brun *et al*., 2015). A phase retrieval preprocessing algorithm was applied before the conventionally filtered back‐projection algorithm to increase the image contrast (Paganin *et al*., 2002). One PCμCT slice per sample was then analyzed with the ImageJ software (see the ImageJ analysis section).

### ImageJ analysis

**Fig. 1:**
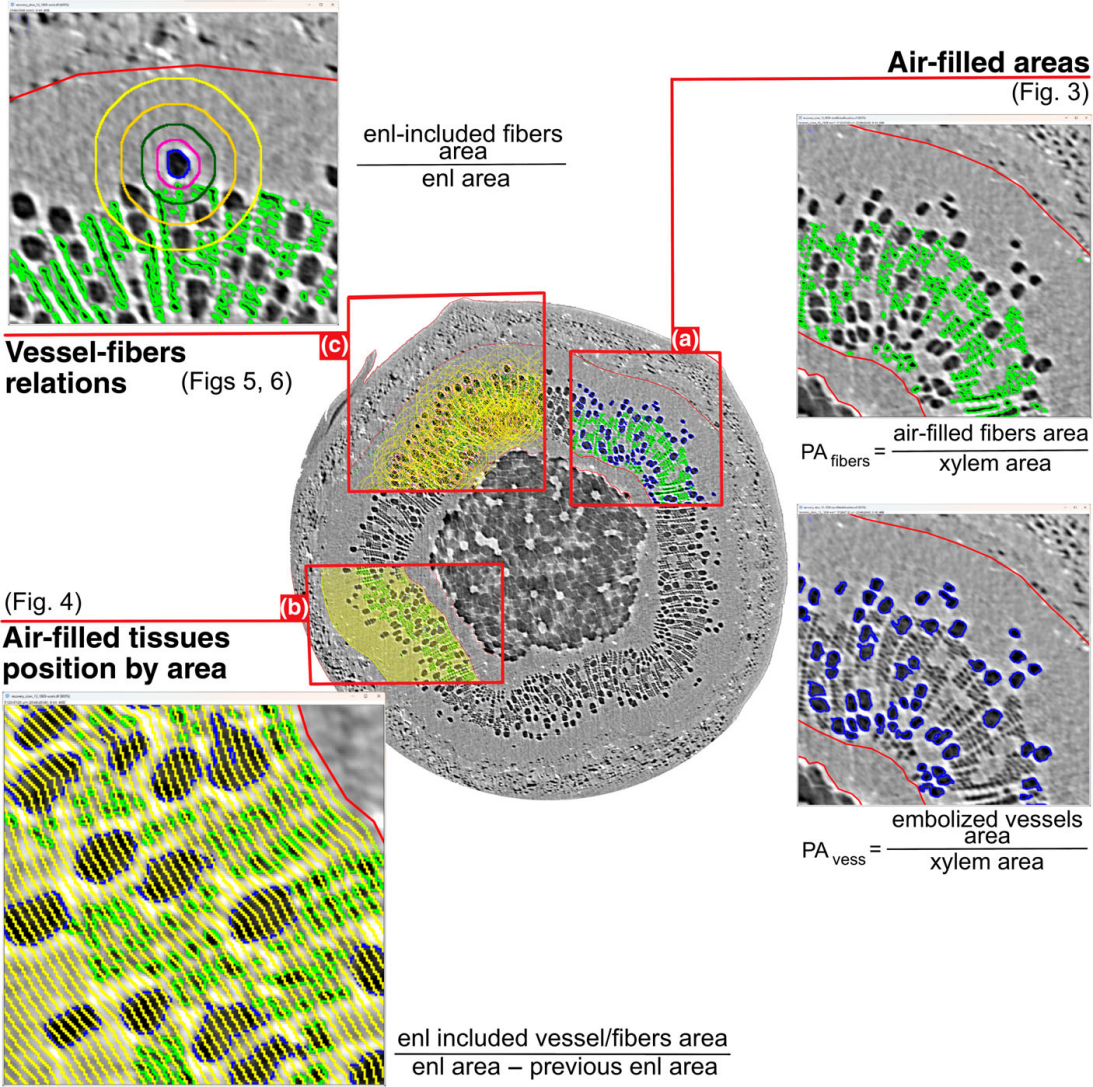
Summary of two‐dimensional image analysis performed on phase‐contrast micro‐computed tomography (PCμCT) slice reconstructions. Enl: enlargement of a region of interest (ROI). Transversal PCμCT section with different ROIs highlighted; blue ROIs indicate embolized vessels, green ROIs indicate air‐filled fibers, whereas red selections point out the total xylem borders. (a) Air‐filled ROIs total area analysis. (b) ROIs spatial distribution analysis by xylem area increments. (c) Single embolized vessels – air‐filled fibers ROIs association analysis. The example poplar stem is part of the recovered from slow‐developed drought group (R_SDD).

Two‐dimensional (2D) PCμCT slices analysis was performed through user‐supervised, semi‐automated routines in ImageJ (Fiji distribution package, ImageJ2 v.1.54f; Schindelin *et al*., 2012). A schematic representation of 2D image analyses performed on PCμ‐CT reconstructed slices is provided in Fig. 1. Nonfunctional vessels were identified on binarized images using the ‘analyze particles’ function that relied on user‐defined circularity and size parameters, in combination with manual observations. Additionally, the areas of the pith and xylem were obtained from the images and used to calculate the percentage of embolized vessel area over the total xylem area (PA_vess_, percentage embolized area of vessels) (Fig. 1a).

$${\mathrm{PA}}_{\text{vess}}=\frac{\text{embolized vessels area}}{\text{total xylem area}}\times 100$$

Water‐depleted fibers were identified as all the remaining embolized cells (by gray value) in the xylem after subtracting the regions of interest (ROIs) previously defined as vessels (PA_fibers_, percentage embolized area of fibers) (Fig. 1a).

$${\mathrm{PA}}_{\text{fibers}}=\frac{\mathrm{air}\;\text{filled fibers area}}{\text{total xylem area}}\times 100$$

Due to the small cross‐sectional area of the fibers, establishing single centroids for each fiber cell recognition was challenging, making any centroid‐based approach (Secchi *et al*., 2021) not precise enough. To address this problem, when measuring the radial arrangement of the air‐filled fibers, we used an area‐based approach by identifying fiber cells as all the pixels outside of the vessel ROI thresholded as embolized. Small amounts of background noise (darker pixels) were likely included in the fibers ROI, appearing in the graph (Fig. 5b, see later) as small values in the outer parts (higher covered percentage) of the xylem.

To assess the radial distribution in the xylem area, the inner xylem ROI was enlarged outward by increments of 10 pixels, ensuring it remained contained within the xylem area. The included xylem area and empty fiber area were then measured. This process was repeated until the entire xylem was covered. This approach enabled us to identify the percentage of fibers relative to different sectors of the xylem. For comparative purposes, the same process was also applied to assess embolized vessels position (Fig. 1b).

Similarly, the presence of water‐depleted fibers near embolized conduits was measured by iteratively processing each single embolized vessel. Each ROI was enlarged by *x* pixels (where *x* = 10, 15, 20, and 25 pixels), and the resulting selection was intersected with the empty fibers ROI (Fig. 1c). This allowed measuring the area occupied by air‐filled tissues near the embolized vessels. For each biological replicate, vessels with water‐depleted fibers nearby (together with the relative area of air‐filled fibers included) and those without were counted.

### Scanning electron microscope anatomical analysis

Two‐centimeter‐long‐stem segments of *P. nigra* were cut 25 cm below the plant apex, similar to the position of X‐ray scan observations. The segments were then hand‐cut with a blade exposing transversal and radial sections and scraped with a sliding Reichert microtome to obtain a smooth surface. Samples were immediately stabilized with double‐coated carbon conductive tabs (PELCO Tabs™) on pin‐mounting stubs (25.4 mm diameter) and transferred to the scanning electron microscope (SEM; Phenom XL G2 Desktop) for imaging. Under the SEM microscope, the anatomical features of vessels and fibers morphology, their cell walls, and connections were observed.

### Data analysis

Some simple calculations were performed in the ImageJ (Schindelin *et al*., 2012) macroscripts. All statistical analyses were conducted using R (R v.4.4.1 (2024‐06‐14 ucrt) (R Core Team, 2022)) in the R Studio environment (Posit team, 2023). ANOVA was carried out and coupled with the Waller–Duncan *post hoc* test (de Mendiburu, 2023) to determine differences between group means. Graphs were generated using the ggplot2 package (Wickham, 2016) and organized using patchwork (Pedersen, 2023). Differences between nonnormally distributed groups were assessed using the Kruskal–Wallis nonparametric test, followed by the Dunn (Ogle *et al*., 2023) *post hoc* test with the Benjamini–Hochberg adjustment. Logit normalization was performed on percentage values to obtain closer to normal distributions before means analysis using the car R package (Fox & Weisberg, 2019).

## Results

### Effects of drought progression rates on plant growth

**Fig. 2:**
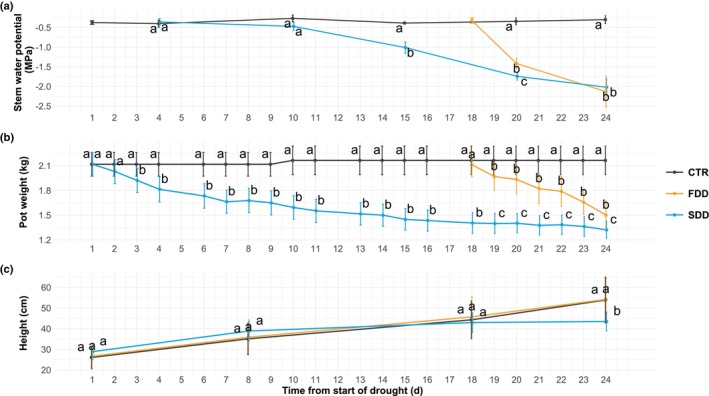
Temporal dynamics during the imposition of different drought rates of: (a) poplar stem water potential; (b) pot weight and plant height. Points represent mean values and bars denote SD. Day 1: start of stress treatment for SDD plants; Day 18: start of treatment for FDD poplars. One‐way ANOVA tests significant differences between the three conditions for each day; different letters denote nonhomogeneous groups (*P* < 0.05) based on the Waller–Duncan method. CTR: well‐watered condition; FDD: fast‐developed drought; SDD: slow‐developed drought.

Two irrigation regimes were implemented to induce Ψ_stem_ values below −2.0 MPa at different rates. The slow developed drought (SDD) induced an average water potential drop rate of 0.09 MPa d^−1^, while the fast‐developed drought (FDD) caused an average rate of 0.34 MPa water potential drop d^−1^. At the end of treatments, the average Ψ_stem_ was −2.13 ± 0.40 MPa for FDD plants and −2.02 ± 0.22 MPa for SDD plants. The well‐irrigated control (CTR) group remained at constant ψ_stem_ values over time (Fig. 2a), at *c*. −0.4 MPa. At the end of the drought period, the pot weights of the two differently stressed groups were slightly (*c*. 250 g) but significantly higher in FDD plants than in SDD plants (Fig. 2b). No difference in growth was observed between CTR and FDD poplars (plant height of 54.2 ± 10.4 cm and 53.9 ± 11 cm, respectively). However, the SDD plants were 20% shorter than the other two groups, with significant differences emerging at stress levels corresponding to Ψ_stem_ < 1.5 MPa (i.e. 19 d of slow stress development; Fig. 2c).

### Phase‐contrast micro‐computed tomography analysis of embolized vessels and water‐depleted fibers in xylem tissue

**Fig. 3:**
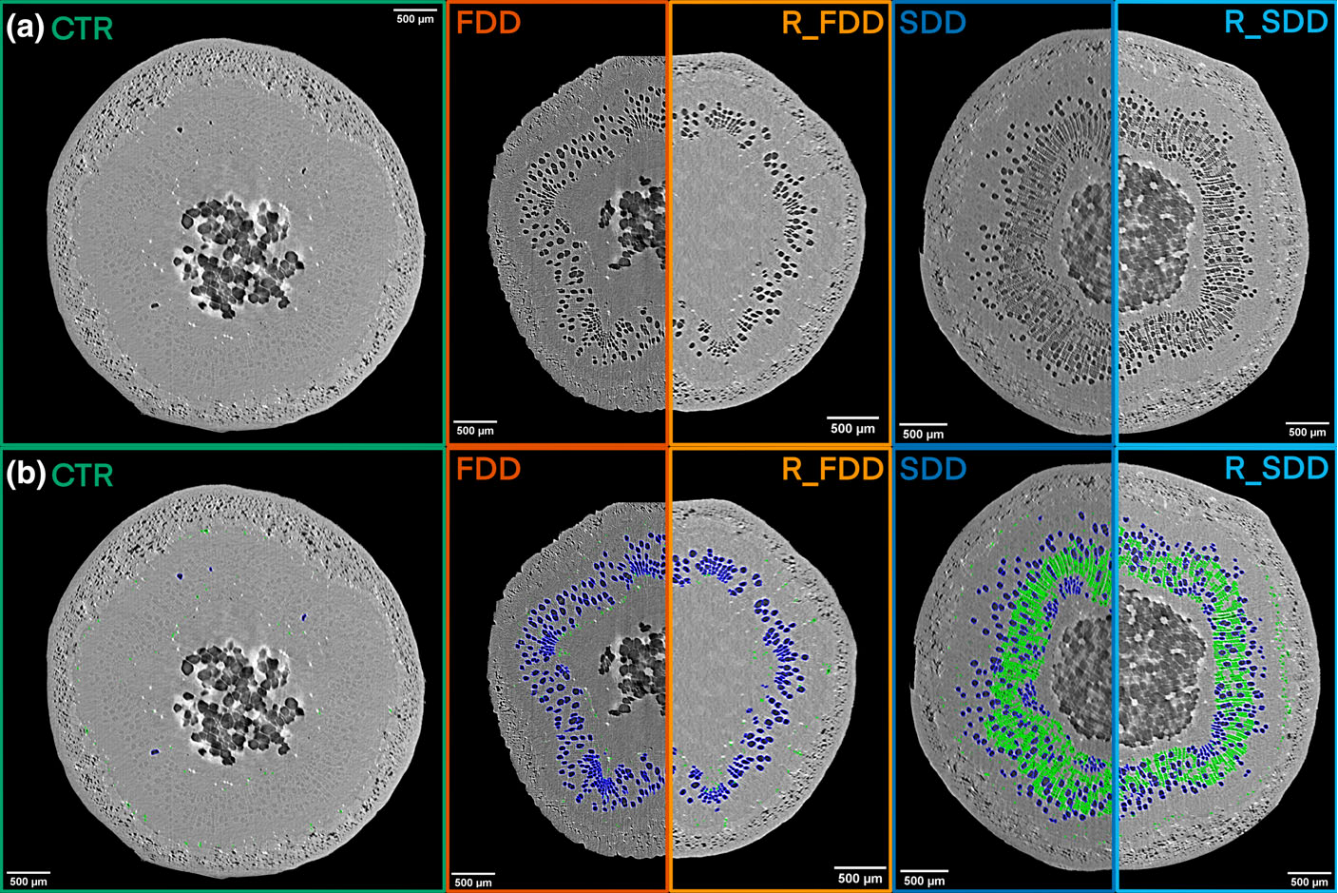
Examples of two‐dimensional (2D) reconstructed *P. nigra* stem images for each treatment. CTR, well‐watered control condition; FDD, fast‐developed drought; R_FDD, recovered fast‐developed drought; R_SDD, recovered slow‐developed drought; SDD, slow‐developed drought. (a) 2D stem reconstructions of an example plant for each treatment. (b) 2D stem reconstructions shown with the fibers (green) and vessels (blue) regions of interest overlay.

**Fig. 4:**
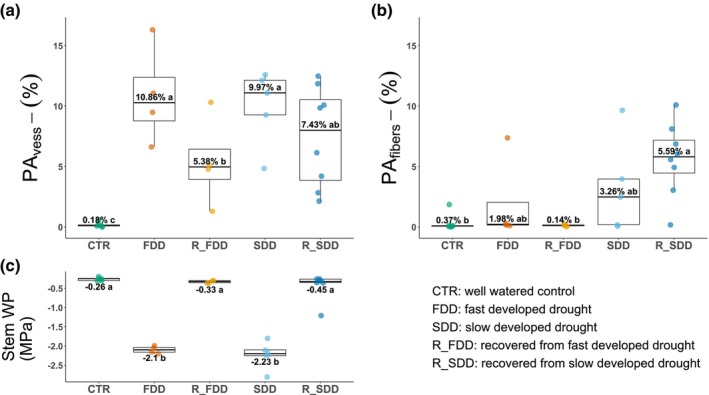
Percentage of embolized vessels and air‐filled fibers area under different conditions. (a, b) Box plot of the percentage of embolized vessels (PA_vess_) and the air‐filled fibers area (PA_fibers_) over total xylem area measured in two‐dimensional reconstructed poplar images of CTR, control; FDD, fast‐developed drought; R_FDD, recovered fast‐developed drought; SDD, slow‐developed drought; R_SDD, recovered slow‐developed drought plants. The line represents the median, and each single point indicate one biological replicate. The boxes represent the interquartile range, while the whiskers indicate values inside 1.5 times the interquartile range from the first and the third quartiles. All points outside the upper/lower fence are outliers. One‐way ANOVA indicates differences in embolized vessels (*P* = 0.0022) and in water‐depleted fibers (*P* = 0.0194). Homogenous groups identified through the Waller–Duncan *post hoc* test are represented by the same letters. (c) Mean stem water potential and boxplot distribution measured before the scansion. Different groups identified using one‐way ANOVA (*P* < 0.0001) followed by the Waller–Duncan *post hoc* test. PA_vess_, percentage embolized area of vessels; PA_fibers_, percentage embolized area of fibers; WP, water potential.

Two‐dimensional PCμCT observations of intact poplar saplings stems revealed that, at the end of drought, the percentage of embolized vessels area over the total xylem area was similar in both groups but significantly higher than in the controls (respectively, FDD: 10.86% ± 4.06; SDD: 9.97% ± 3.15; CTR: 0.18% ± 0.21) (Figs 3, 4a). While slow‐ and fast‐stressed plants presented similar values of final PA_vess_ (percent embolized vessels area), PA_fibers_ was higher in SDD poplars (3.26% ± 3.92) than in FDD plants (1.98% ± 3.58), even though this difference was not statistically significant (Fig. 4b). This could be attributed to one notable outlier in the FDD group, which showed values of the area occupied by gas‐filled fibers below 0.3% for all the replicates except for one sample (7.35%). Independently of the condition, in the same stem section, the larger area of embolized vessels was not associated with a higher area of empty fibers, suggesting that the formation of vessel embolism did not influence the occurrence of water depletion in fibers. Almost no gas‐filled fibers were detected in stems of CTR plants (Figs 3, 4b).

Slow‐developed drought and FDD‐stressed poplars were then rewatered to their pot capacity and allowed to recover over a 24‐h period. After this time interval, Ψ_stem_ in both groups returned to values similar to controls, as average Ψ_stem_ was −0.26 ± 0.04 MPa for CTR plants, −0.33 ± 0.03 MPa for R_FDD, and −0.45 ± 0.37 MPa for R_SDD (Fig. 4c). Despite complete Ψ_stem_ recovery, only a partial restoration of vessel hydraulic functionality was observed in both plant groups (Fig. 4a) during the allotted time. However, the R_FDD group presented a significant reduction in PA_vess_ by over 50%, and the extent of embolism was lower than that observed for FDD plants. R_SDD plants exhibited a reduction in PA_vess_ by *c*. 25%, which was not significantly different from SDD plants (Fig. 4a). After rewatering, only a few water‐depleted fibers were observed in R_FDD plants, with PA_fibers_ values similar to CTR and FDD plants (FDD vs R_FDD *P* = 0.380). On the other hand, after stress relief, R_SDD poplars showed PA_fibers_ values similar to SDD plants (SDD vs R_SDD *P* = 0.294) and significantly higher than R_FDD ones (Fig. 4b).

### Spatial distribution of embolized vessels and water‐depleted fibers

**Fig. 5:**
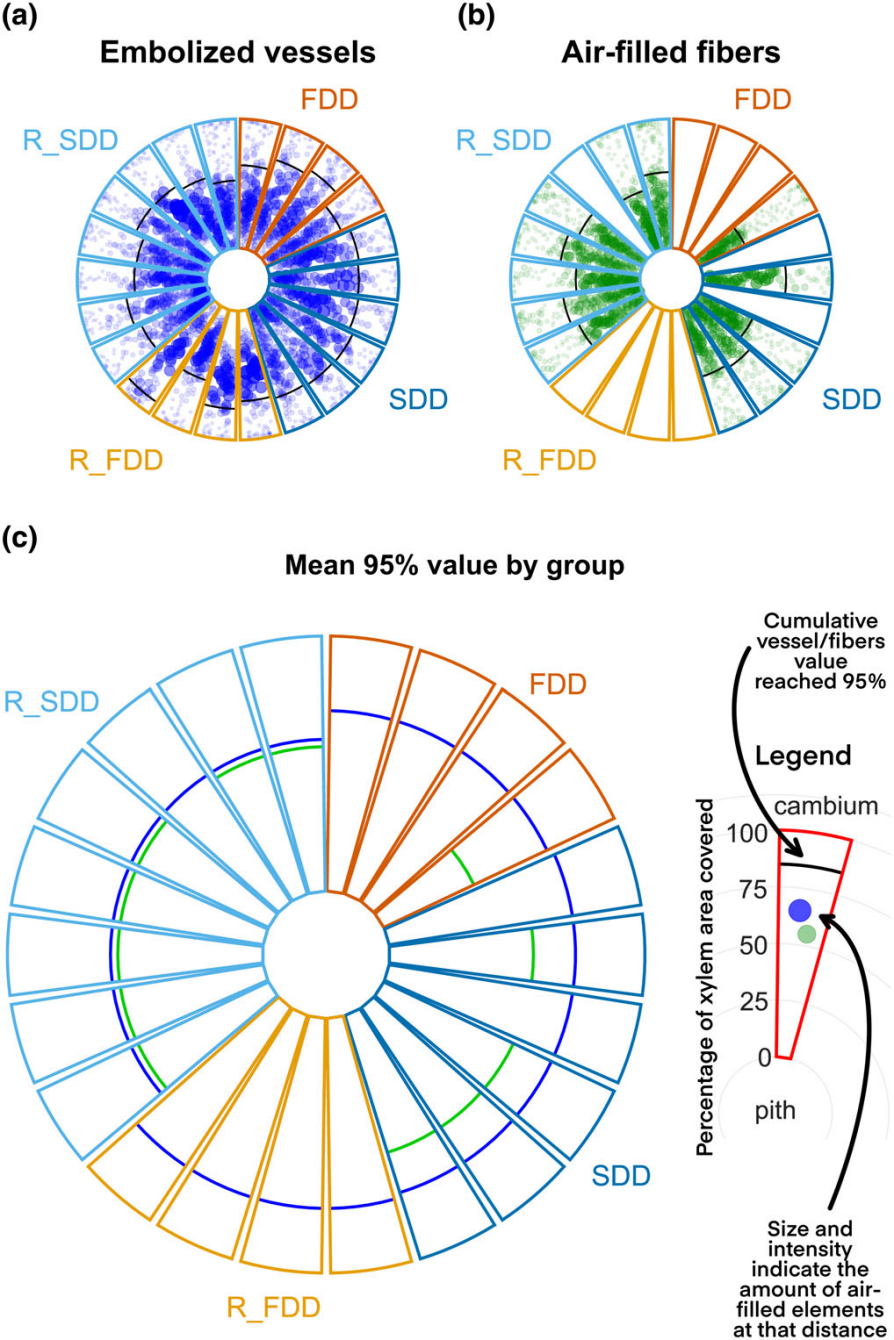
Spatial distribution of embolized vessels and air‐filled fibers. (a, b) Amount of embolized vessels (blue dots) and water‐depleted fibers (green dots) percentage by position in the xylem. Each slice represents a single biological replicate; poplar plants with total air‐filled fiber areas under 0.5% were not measured and are presented empty. Point size and intensity indicate the percentage of air‐filled elements at that distance. Distance is represented as a percentage of the covered xylem area. The black line indicates when the cumulative area of the air‐filled component reached the 95% threshold. (c) Mean distance of the 95% threshold for each group when available. Blue lines indicate vessels, while green ones indicate fiber values. FDD, fast‐developed drought; SDD, slow‐developed drought; R_FDD, recovered fast‐developed stress; R_SDD, recovered slow‐developed stress. Mean variance analysis with one‐way ANOVA (*P* = 0.0032) followed by the Waller–Duncan *post hoc* test (SDD: a, FDD: a, R_FDD: a, R_SDD: b).

Image analyses revealed that embolized vessels and air‐filled fibers were mostly located toward the pith, with 95% of embolized vessels located on average within the first 68.0% of xylem area (Fig. 5a) and with 95% of air‐filled fibers on average within the first 55.1% of xylem area (Fig. 5b). It is important to notice that R_SDD plants exhibited a significantly different pattern of embolized vessel distribution when compared to all other treatments, with 95% of embolized vessels located within the first 59% of xylem area (Fig. 5a,c).

### Association between embolized vessels and water‐depleted fibers

**Fig. 6:**
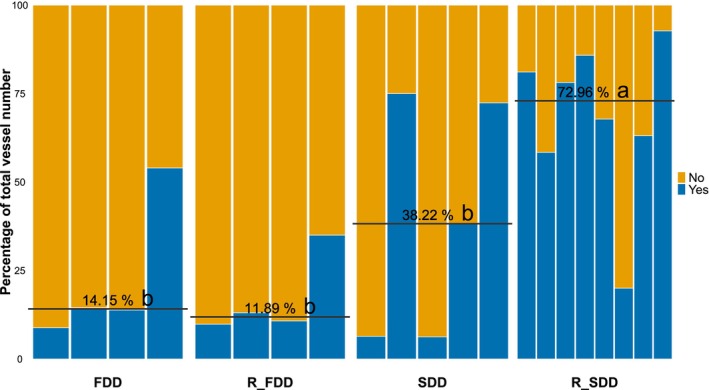
Percentages of embolized vessels surrounded by water‐depleted fibers (Yes, blue bars) and by water‐filled fibers (No, orange bars) in the selected enlargement area. Each condition is represented by single poplar biological replicates (single bar), and median values for Yes are reported. The data are from one enlargement (10 pixels) of the vessel region of interest for assessing the surrounding area in the graph. Different letters indicate different groups, assessed using one‐way ANOVA (*P* = 0.0127) followed by the Waller–Duncan *post hoc* test on logit normalized values. FDD, fast‐developed drought; R_FDD, recovered fast‐developed drought; R_SDD, recovered slow‐developed drought; SDD, slow‐developed drought.

additional image analysis on scanned stem reconstructions was performed in a selected neighboring area (*x* pixel enlargement, see Fig. 1c) to quantify the percentages of embolized vessels surrounded by (1) water‐depleted fibers (YES) and (2) only hydrated fibers (NO). The results revealed that SDD and R_SDD poplars had, on average, a higher number of vessels surrounded by air‐filled fibers than those measured in FDD plants (Fig. 6). Furthermore, the stems of R_SDD plants had almost twice the number of embolized vessels surrounded by empty fibers compared with the SDD group. This increased percentage in the R_SDD group (Fig. 6) was probably caused by the refilling of vessels with water‐filled fibers nearby (*c*. 25% reduction, Fig. 4b), which increased the proportion of embolized vessels with empty fibers that apparently did not recover at the 24‐h time point.

**Fig. 7:**
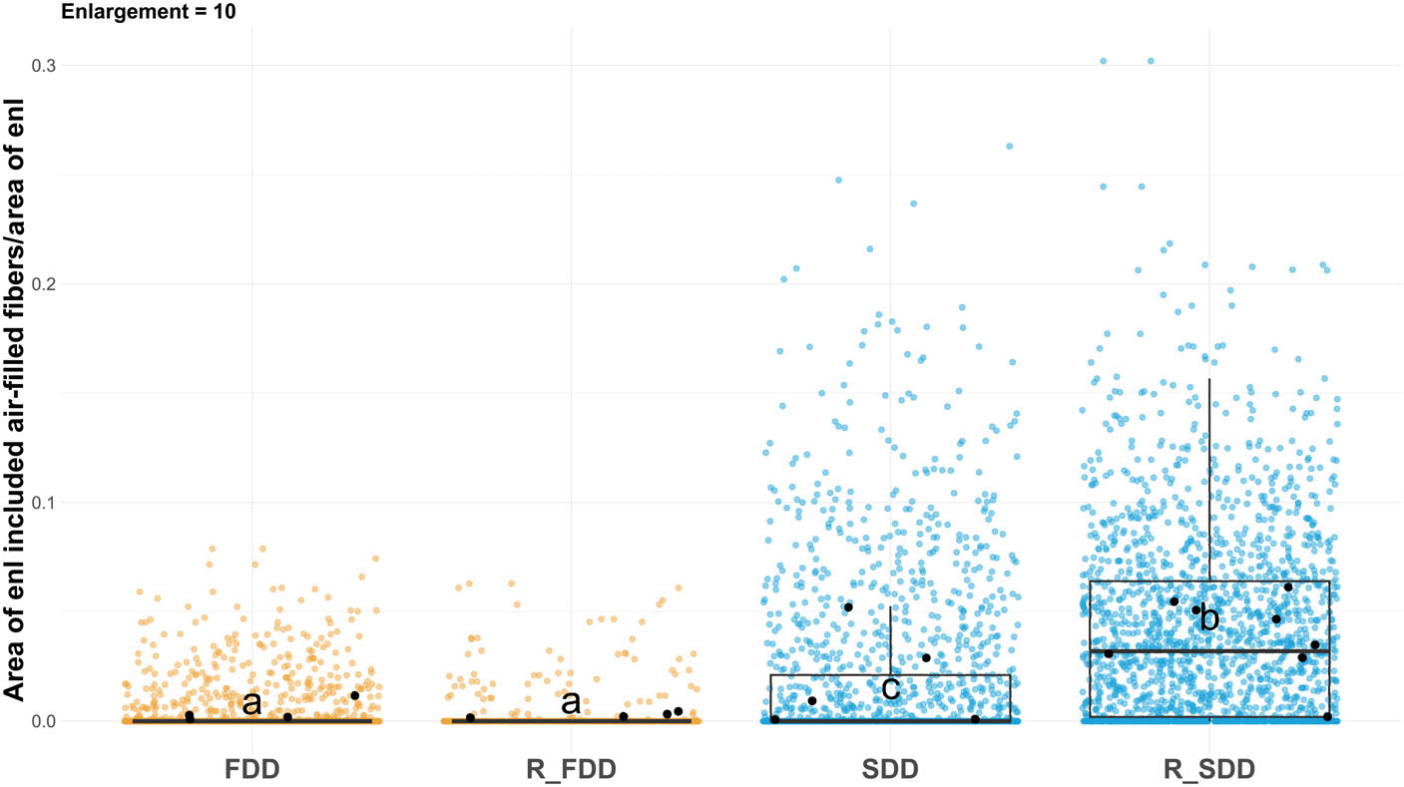
Amount of air‐filled fibers near embolized vessels. Points indicate values of air‐filled fibers area over the enlargement (10 pixels) area around each embolized vessel. Colored points indicate single vessel values, and the boxplots represent their distribution, whereas black points indicate poplar biological replicates median values. In the boxplot, the line represents the median and the box indicates the interquartile range, while the whiskers indicate values inside 1.5 times the interquartile range from the first and the third quartiles. All points outside the upper/lower fence are outliers. Different letters indicate different groups assessed using the Dunn Kruskal–Wallis (*P* < 0.0001) multiple comparison *post hoc* test, *P*‐values adjusted with the Benjamini–Hochberg method (significance at *P* < 0.005). FDD, fast‐developed drought; R_FDD, recovered fast‐developed stress; R_SDD, recovered slow‐developed stress; SDD, slow‐developed drought.

Coherently, the ratio between the areas of air‐filled fibers surrounding the embolized vessels (within 10‐pixel distance from vessel) showed that the amount of air‐filled fibers was significantly higher in SDD and R_SDD plants than in the FDD groups (Fig. 7). Moreover, while no statistically significant difference was found between the FDD and R_FDD groups, the R_SDD plants had a significantly higher amount of empty fibers nearby embolized vessels than SDD ones (Fig. 7), despite similar PA_vess_ (Fig. 4a).

As expected, with the increase in the considered area around the embolized conduits containing water‐depleted fibers, the amount of empty fibers close to gas‐filled vessels also increased (Supporting Information Fig. S1).

Despite that, the significant differences observed among groups in the 10‐pixel enlargement (Figs 6, 7) were confirmed for any enlargements (15, 20, and 25 pixels area, Figs S1, S2).

### Poplar stem anatomical features

**Fig. 8:**
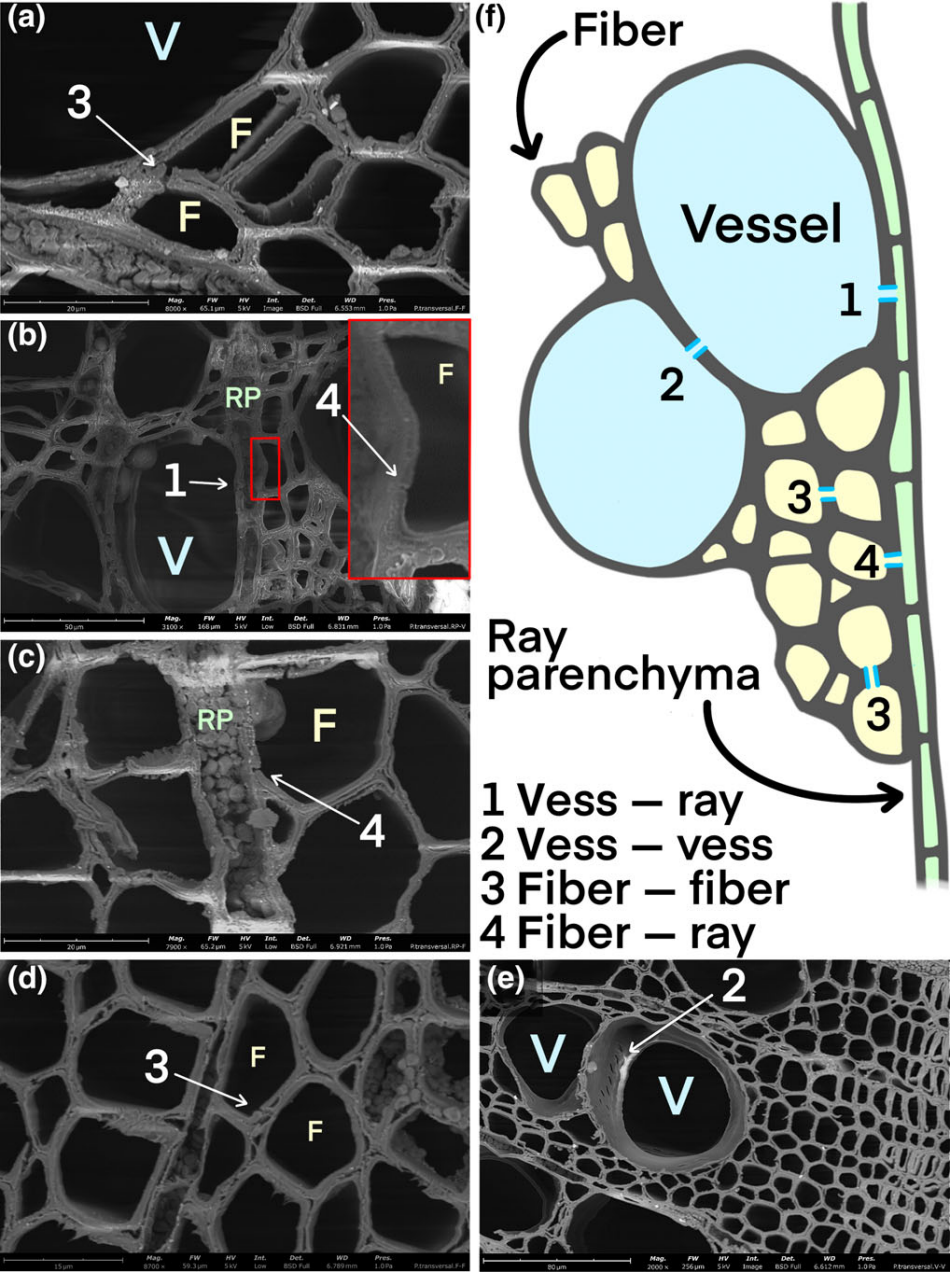
Scanning electron microscopy images of *Populus nigra* xylem. (a–e) arrows indicate pit connections. (f) Schematic representation of poplar xylem element connections in a transverse view based on the anatomical observations (F, fiber; V, vessel; RP, ray parenchyma).

Scanning electron microscope stem cross‐sections showed typical diffuse‐porous xylem with a uniform distribution of vessels immersed in a matrix of fibers and uniseriate parenchyma rays that connect the pith to the phloem (Fig. S3a). Axial parenchyma is almost absent (Fig. S3b). The vessel elements are axially interconnected through simple perforation plates and by radial and tangential intervessel pits (Figs 7, S3b). Similarly, mature poplar fibers exhibit simple pits (*c*. 5 μm wide) on both tangential and radial walls, which are more frequent toward fiber tips (Fig. 8a,d). No pits were observed in either direction between fibers and vessels. Anatomical observations of parenchyma ray cells revealed pits in the direction of fibers (Fig. 8b,c). These pits are simple and extend across the whole secondary wall, from the cell lumen to the middle lamella. The pit channel is uniform in size, but its innermost part, in the proximity of the middle lamella, consistently widens, giving the pits a characteristic ampulla‐like shape in cross‐section. Connections can also be observed between vessels and parenchyma ray cells (Fig. 8b).

## Discussion

### Stress kinetics affect plant growth

Potted *P. nigra* plants were forced to develop similar water stress levels over either a short (*c*. 1 wk; typical pot drought experiments, Pagliarani *et al*., 2019) or long (4 wk; more closely resembling field dynamics, Romero *et al*., 2017) time interval, resulting in different rates of drought progression that affected plant growth. In the first case, drought was imposed by ceasing the irrigation, and within 2 d, the stem water potential dropped by *c*. −1.1 MPa. Conversely, long‐term stress development was induced by gradually reducing the amount of water available, and the xylem pressure dropped by *c*. −1.4 MPa over 20 d. Slow‐developed drought plants exhibited no significant height difference during the first 18 d; however, they did not show any further growth during the last 6 d. Consequently, their height was reduced compared with well‐irrigated poplars by the end of the treatment period. Conversely, plants subjected to a FDD did not have time to modulate their growth; they continued to increase in height until the end of the experimental trial.

### Stress kinetics do not impact the level of drought‐induced embolism, but influence the recovery

Phase‐contrast micro‐computed tomography reconstructed sections of stems of intact poplars revealed that the water stress level, but not the rate at which stress was imposed, affects the final level of vessel embolism. Percentage embolized area of vessels values were close to those measured previously in poplars (*P. tremula × alba*) exposed to a fast drought (Secchi *et al*., 2021) and were significantly higher than in the control group. Interestingly, a 24‐h recovery period resulted in a notable reduction in PA_vess_ in plants exposed to fast drying (by *c*. 50%), while recovery in plants exposed to slow drying was much less pronounced (*c*. 25%), with the final level of embolism not being significantly different from that of SDD plants. The partial hydraulic recovery observed in this study is consistent with that observed previously *in vivo* or through classical hydraulic measurements on poplars, underlining that full restoration of stem hydraulic capacity occurs only after several days (Pagliarani *et al*., 2019; Secchi *et al*., 2021; Rosso *et al*., 2023). Similar results were reported also for *Laurus nobilis* L. plants exposed to long‐term water shortage, which showed a lower ability to recover from xylem embolism 24 h after rewatering compared with plants exposed to short‐term drought. A full recovery was observed only after 7 d of irrigation (Trifilò *et al*., 2017).

Embolism formation and its recovery have been observed to be spatially coordinated with embolism progression initiating near the pith and expanding toward the cambium as tension increases and the recovery occurring from the cambium toward the pith (Brodersen & McElrone, 2013; Wang *et al*., 2013; Choat *et al*., 2016; Secchi *et al*., 2021). In this study, we quantified the spatial distribution of embolized vessels and confirmed that vessels most likely embolized from pith outward, as 95% of embolized vessels were located within the first 68% of the distance between pith and cambium.

The recovery pattern was not as clear. A general reduction of embolized vessels across the stems was observed in R_FDD plants, but no difference in their distribution was detected. In fact, 95% of the embolized vessels were located within the first 78.8% and 74.6% of the distance between pith and cambium for FDD and R_FDD plants, respectively. Conversely, an outward‐to‐inward recovery was observed in R_SSD, as supported by the shift of 95% of embolized vessels from 72.8% to 59.6% of the distance between pith and cambium, indicating that the vast majority of embolized vessels were, on average, 13.2% closer to the pith.

### Stress kinetics impact the amount of empty fibers but not recovery

The amount of water‐depleted fibers differed between the two groups at the end of stress treatments. Plants subjected to FDD exhibited a nearly negligible presence of air‐filled fibers, while stems of plants exposed to SDD displayed high levels of air‐filled fibers. These results are consistent with previous observations on intact potted *P. alba × tremula* plants exposed to fast dehydration, where no or very low levels of empty fibers were detected (Secchi *et al*., 2021). No air‐filled fibers were also found in stems of *P. nigra* poplars subjected to an FDD‐like treatment (Tomasella *et al*., 2021). After stress recovery, there were no significant changes in the amount or spatial distribution of empty fibers.

Similar to vessels, empty fibers were located mostly close to the pith, with 95% of them located within *c*. 55% of the distance between pith and cambium. This spatial distribution can be attributed, as reported by Courtois‐Moreau *et al*. (2009), to the different phases of autolytic processes resulting in poplar fiber cell death. Indeed, fiber cell death occurs later than vessel death, and the complete dismantling of cellular remnants may require additional time. Although the vast majority of fibers in our sections were structurally mature with complete secondary wall deposition (Fig. S4a), we hypothesize that fibers in the outer part of the xylem, close to the cambium, might still be filled with cellular remnants rather than water (micro‐CT observations do not allow to distinguish between these two situations) (Fig. S4b). Consequently, in this region, they never appeared empty.

The observed presence of empty fibers in plants exposed to slow drying (SDD) underlines the importance of applying field‐relevant stress conditions during drought experiments, considering that the progression of stress impacted fiber water depletion more than the final and absolute water stress level. For example, empty fibers were not observed in previous experiments (Brodersen *et al*., 2013, 2018; Losso *et al*., 2019; Vuerich *et al*., 2023), but this could likely be due to the common practice of rapid stress imposition experimental setups, in addition to species‐specific differences in xylem anatomy.

### Recovery from embolism—new insight

We observed different levels of vessel embolism recovery between R_FDD and R_SDD poplar plants. Recovery can occur only if a free water source is available. Typically, it is assumed that refilling water is provided by the phloem via radial parenchyma, but no direct evidence was provided in this respect. Interestingly, a comprehensive study on 30 angiosperm species, aimed at estimating water discharge from the xylem in excised stems, indicated that free water was drawn from fibers during daily changes in water potential (Ziemińska *et al*., 2020). Similarly, imperforated tracheary elements were shown to alleviate xylem tension in a dehydration setup (Yazaki *et al*., 2020). In both studies, direct connections between vessels and dead fibers would be required to create hydraulic continuity between the two lumina, allowing water flow during changes in water demand. In this model, fibers act as a volume that can buffer the water requirement during changes in vessel tension. However, the role of fibers in the recovery process seems unlikely.

In this study, PCμCT observations highlighted that dead fibers did not reduce the risk of drought‐induced vessel embolism by releasing water into the transpiration stream. Indeed, stems with air‐filled fibers (SDD plants) exhibited a similar percentage of vessel embolism as stems with only water‐filled fibers (FDD plants). Although the dynamics of water depletion in fibers during SDD were not specifically tracked, our results suggest that fibers might indirectly contribute to the recovery of embolized vessels. Specifically, we observed that in R_SDD stems, 24 h after stress relief, there was a significant increase in the proportion of embolized vessels associated with a high number of empty fibers compared to SDD plants. This finding suggests that vessels with few or no air‐filled fibers in their surroundings (as in the case of R_FDD plants) were the first to recover from embolism, whereas embolized vessels close to empty fibers were less likely to recover their functionality. These observations indicate that the presence of water‐filled fibers around the embolized vessels can enhance their chances of refilling.

### Fibers water release and usage through parenchyma cells

Recently, Knipfer *et al*. (2019) pointed out a possible functional role of mature fibers associated with xylem anatomical features and pit connections. In chestnut stems, the fibers network is separated from the vessel lumen by the presence of living parenchyma cells. Thus, the water released from fibers can be redistributed within the stem and taken up by living cells to maintain cambial cell vitality, which is crucial for stress recovery and the resumption of growth. Similarly, our anatomical analysis revealed that *P. nigra* stems do not have direct connections between vessels and dead fibers, whereas connections were observed, surprisingly, between fibers and ray parenchyma cells, as well as between vessels and living cells. This anatomical arrangement suggests that water movement from fibers to vessels might be mediated by living cells. This is in line with the body of evidence indicating vessel‐associated parenchyma as the immediate source of water for the refilling of the conduits (Brodersen & McElrone, 2013; Brodersen *et al*., 2018; Secchi *et al*., 2021).

**Fig. 9:**
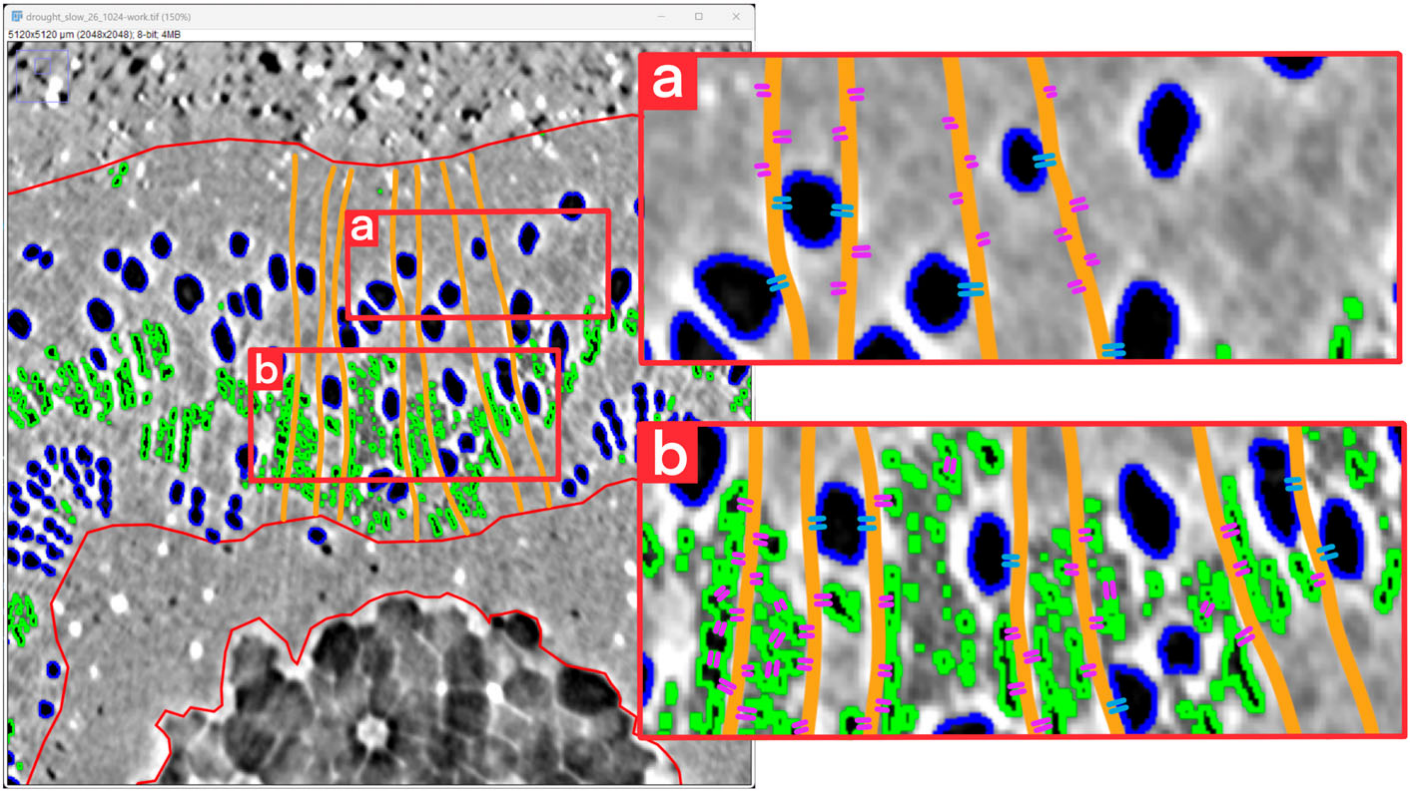
Model of proposed roles of fibers during prolonged water stress and recovery based on connections on reconstructed PCμCT‐CT section of intact poplar stems. Vessels are highlighted in blue, fibers in green. Orange lines indicate the hypothetical position of parenchyma rays. Pink lines highlight possible connections for water movement from fibers, while vessel‐ray piths are in light blue. (a) Details of hypothetical embolized vessel – water‐filled fibers – parenchyma ray connections; (b) details of hypothetical embolized vessel – water‐depleted fibers – parenchyma ray connections.

Based on our results, we propose a model for poplar xylem (Fig. 9) hypothesizing a role for fibers during extended water stress and recovery. During prolonged periods of drought, fibers act as water reserves for living tissues, as water can be actively drawn out by parenchyma cells. In our model, water released from fibers does not lower the risk of vessel embolism, and instead, it is used to maintain cell hydration. Fiber water volumes are relatively small compared with nearby vessels and are unlikely to account for increased water demands under prolonged stress. However, they are potentially sufficient to ensure minimal hydration of living cells, thereby guaranteeing their survival. Furthermore, we observed radial connections between fibers, indicating that direct radial spread of gas is possible in poplar fibers. Consequently, the radial disposition of air‐filled fibers in our samples may be attributed both to water redistribution and to the positioning of parenchyma rays, often observed bordering the air‐filled fibers (Fig. 9).

Our *in vivo* observations showed that:

- after prolonged stress, a significant amount of fibers was air‐filled;
- the fibers closer to the pith can be water‐depleted, whereas those located in the outer section of the xylem, near the cambium, are almost never air‐filled. This is probably due to the different phases of autolytic processes resulting in poplar fiber cell death;
- axial parenchyma is almost absent in this poplar species, and ray parenchyma cells represent a small fraction of xylem, thus providing a relatively limited volume of water;
- parenchyma ray cells are connected via pits with the lumina of both vessels and fibers, whereas vessels and fibers are never connected;
- after re‐watering, in R_SDD stems the distribution of empty fibers did not change, and they occupied *c*. 54% of the total xylem area close to the pith. On the other hand, after stress relief, the distribution of the nonfunctional conduits changed, moving closer to the pith in the area where the water‐depleted fibers are mostly distributed. This suggests that the first vessels to recover are those located farther from empty fibers;
- after stress relief, R_SSD plants recovered only 25% of embolism, and vessels surrounded by water‐filled fibers were more likely to recover.

Based on these observations, we suggest that during recovery from drought, ray parenchyma cells not only actively supply water to empty conduits (Brodersen & McElrone, 2013; Secchi *et al*., 2017) but also provide a pathway for moving water from fibers. This ensures hydration of living tissues under prolonged stress and facilitates the restoration of hydraulic conductivity when tension is reduced (Fig. 9). The possible role of fibers in facilitating recovery is also supported by *in vivo* analysis on R_FDD intact stems, which contained water‐filled fibers and exhibited a much higher rate of embolism recovery compared to R_SDD plants.

If the fibers provide water to the parenchyma to facilitate the refilling of embolized vessels, then it is reasonable to expect them to empty during the recovery process. However, our *in vivo* observations did not detect this pattern, suggesting that the hydration state of the dead fibers is likely maintained during recovery. Coherently, anatomical analysis performed on young poplar shoots (*Populus euramericana*) revealed that wood cross‐sectional area was occupied by 23% vessel lumina and 28% fibers lumina, indicating that fibers can potentially hold similar amounts of water as the vessels (Jourez *et al*., 2001). From our anatomical analysis, we observed that the fibers lumina surface that on average embolized (*c*. 55%) is comparable to the parenchyma area (parenchyma area/fibers area = 0.75, Fig. S4). These values suggest that the water contained in the fibers can have a strong impact on parenchyma hydration levels during prolonged stress. Furthermore, we hypothesize that the availability of water nearby embolized conduits is advantageous in the immediate period after recovery to kick‐start the refilling process, whereas new water arrives to compensate for the remobilized one, thereby facilitating recovery in plants experiencing fast‐developing stress. Although the impact of fibers in fully grown plants, where the developing xylem sections are proportionally insignificant, remains unknown, they may play an increasingly important role as a water reserve. Under natural/agricultural environmental conditions, plants frequently encounter intense stress events. Our findings suggest that rather than intensity, the progression rate of drought has the largest impact on plants capacity to cope with and recover from hydraulic damage. Thus, plants exposed to stress dynamics resembling field conditions may struggle to recover, as their fiber water reserves become depleted during the stress, reducing their recovery capacity and, over time, this could compromise their survival.

## Competing interests

None declared.

## Author contributions

FS and MAZ conceived the study and designed the experiments. FS and SC performed the stress experiments. FS, MT, SC, FP, SN, LD, GT, AN and MAZ were involved in PCμCT observations. NT and FS performed the image reconstruction and image analysis. NT wrote the ImageJ and R code. AC, RG and AP performed the anatomical analysis. NT, FS, AN and MAZ contributed to the analyses and discussion of data. NT, FS, MAZ and AN wrote the manuscript, with contributions and revisions. FS and MAZ contributed equally to this work.

## Disclaimer

The New Phytologist Foundation remains neutral with regard to jurisdictional claims in maps and in any institutional affiliations.

## Supporting information


**Fig. S1** Percentages of embolized vessels surrounded by water‐depleted fibers and by water‐filled fibers nearby in the selected enlargement area.
**Fig. S2** Amount of water‐depleted fibers at different distances from embolized vessels.
**Fig. S3** Additional SEM images.
**Fig. S4** *Ex vivo* xylem anatomy.
Please note: Wiley is not responsible for the content or functionality of any Supporting Information supplied by the authors. Any queries (other than missing material) should be directed to the *New Phytologist* Central Office.

## Data Availability

Data available in article (Figs S1–S4).
