# Supplementary material for: Fibers beyond structure: do they contribute to embolism reversal after drought relief in poplar?
Source: New Phytol. 2025 May 1;247(2):612–24. doi: 10.1111/nph.70179 (PMC12177288; doi:10.1111/nph.70179)
Supplement: Supplementary file 1 — Fig. S1 Percentages of embolized vessels surrounded by water‐depleted fibers and by water‐filled fibers nearby in the selected enlargement area. Fig. S2 Amount of water‐depleted fibers at different distances from embolized vessels. Fig. S3 Additional SEM images. Fig. S4 Ex vivo xylem anatomy. Please note: Wiley is not responsible for the content or functionality of any Supporting Information supplied by the authors. Any queries (other than missing material) should be directed to the New Phytologist Central Office. [file NPH-247-612-s001.pdf]

New Phytologist

New Phytologist Supporting Information

Article title: Fibers beyond structure: Do they contribute to embolism reversal after drought relief in poplar?

Authors: Niccolò Tricerri, Martina Tomasella, Silvia Cavalletto, Francesco Petruzzellis, Sara Natale, Alan Crivellaro, Rachele Gamba, Alma Piermattei, Lorenzo D'Amico, Giuliana Tromba, Andrea Nardini, Maciej A. Zwieniecki, Francesca Secchi

Article acceptance date: 4 April 2025

The following Supporting Information is available for this article:

Fig. S1 Percentages of embolized vessels surrounded by water-depleted fibers and by water-filled fibers nearby in the selected enlargement area.

Fig. S2 Amount of water-depleted fibers at different distances from embolized vessels.

Fig. S3 Scanning Electron Microscopy images of stem cross-section, showing (a) whole cross section and (b-c-d) xylem details. F, fiber; V, vessel; RP, ray parenchyma, VAC, vessel associated cells.

Fig. S4 Ex-vivo xylem anatomy.

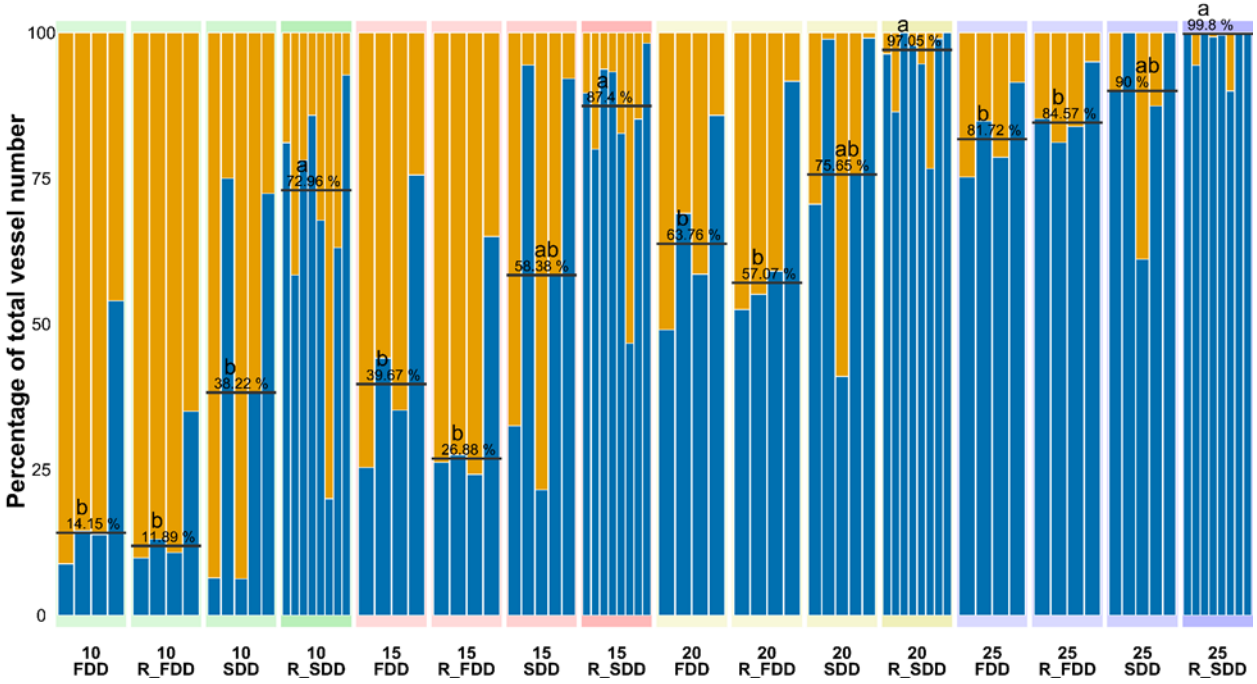

Fig. S1 Percentages of embolized vessels surrounded by water-depleted fibers (YES, blue bars) and by water-filled fibers nearby (NO, orange bars) in the selected enlargement area.
Each condition is represented by single biological replicates (single bar), and median values for YES are reported. The data is from all enlargements performed (10, 15, 20, 25px) of the vessel region of interest for assessing the surrounding area in the graph. Different letters indicate different groups, assessed using one-way ANOVA followed by Waller-Duncan post hoc on logit-normalized values. FDD: fast developed drought, SDD: slow developed drought, R_FDD recovered fast developed stress, R_SDD recovered slow developed stress.

[[10]] p.value 0.0112 *
[[15]] p.value 0.0143 *
[[20]] p.value 0.0173 *
[[25]] p.value 0.0112 *

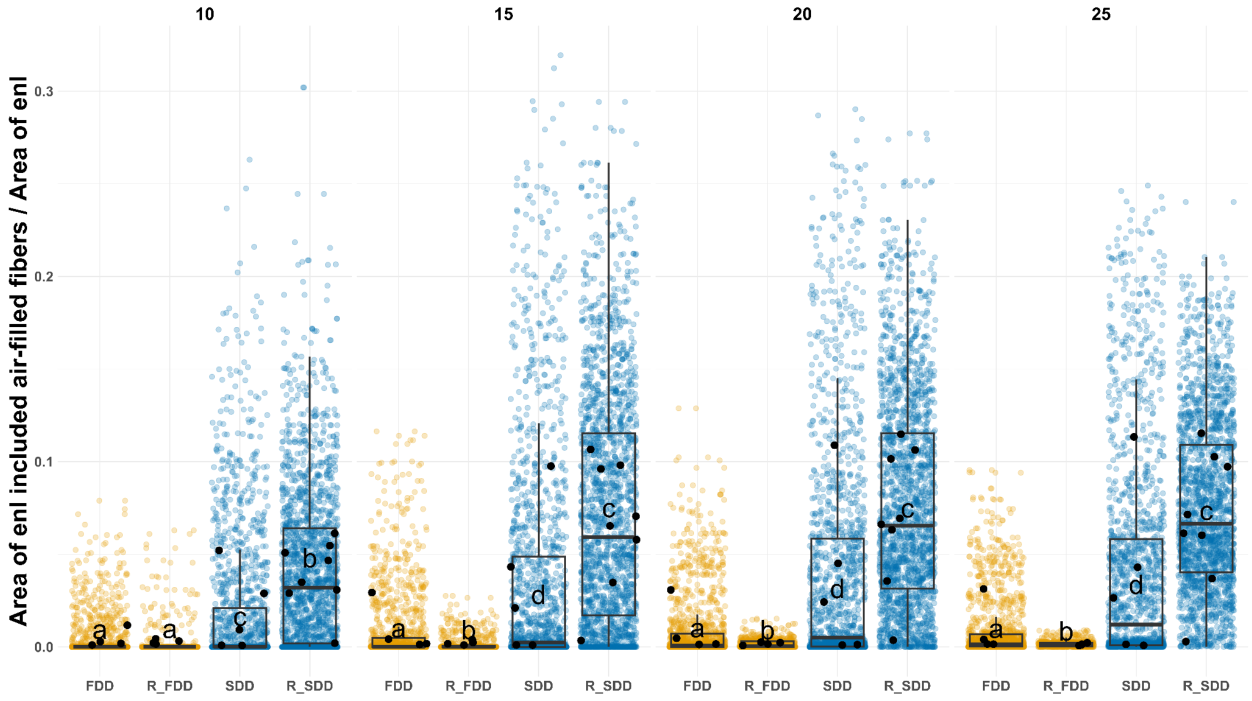

Fig. S2 Amount of water-depleted fibers at different distances from embolized vessels. Points indicate values of air-filled fibers area over the enlargement (values 10, 15, 20, 25 px) area around each embolized vessel. Colored points indicate single vessels values and the boxplots are representing their distribution while black points indicate biological replicates median values. Different letters indicate different groups assessed using the Dunn Kruskal-Wallis ($p < 0,0001$) multiple comparison post hoc test, p-values adjusted with the Benjamini-Hochberg method (significance at $p < 0.005$). FDD: fast developed drought, SDD: slow developed drought, R_FDD recovered fast developed stress, R_SDD recovered slow developed stress.

[[10px]] Kruskal-Wallis rank sum test. Kruskal-Wallis chi-squared = 1539.5, df = 3, p-value < 2.2e-16

[[15px]] Kruskal-Wallis rank sum test. Kruskal-Wallis chi-squared = 1782.5, df = 3, p-value < 2.2e-16

[[20px]] Kruskal-Wallis rank sum test. Kruskal-Wallis chi-squared = 2157.9, df = 3, p-value < 2.2e-16

[[25px]] Kruskal-Wallis rank sum test. Kruskal-Wallis chi-squared = 2397.5, df = 3, p-value < 2.2e-16

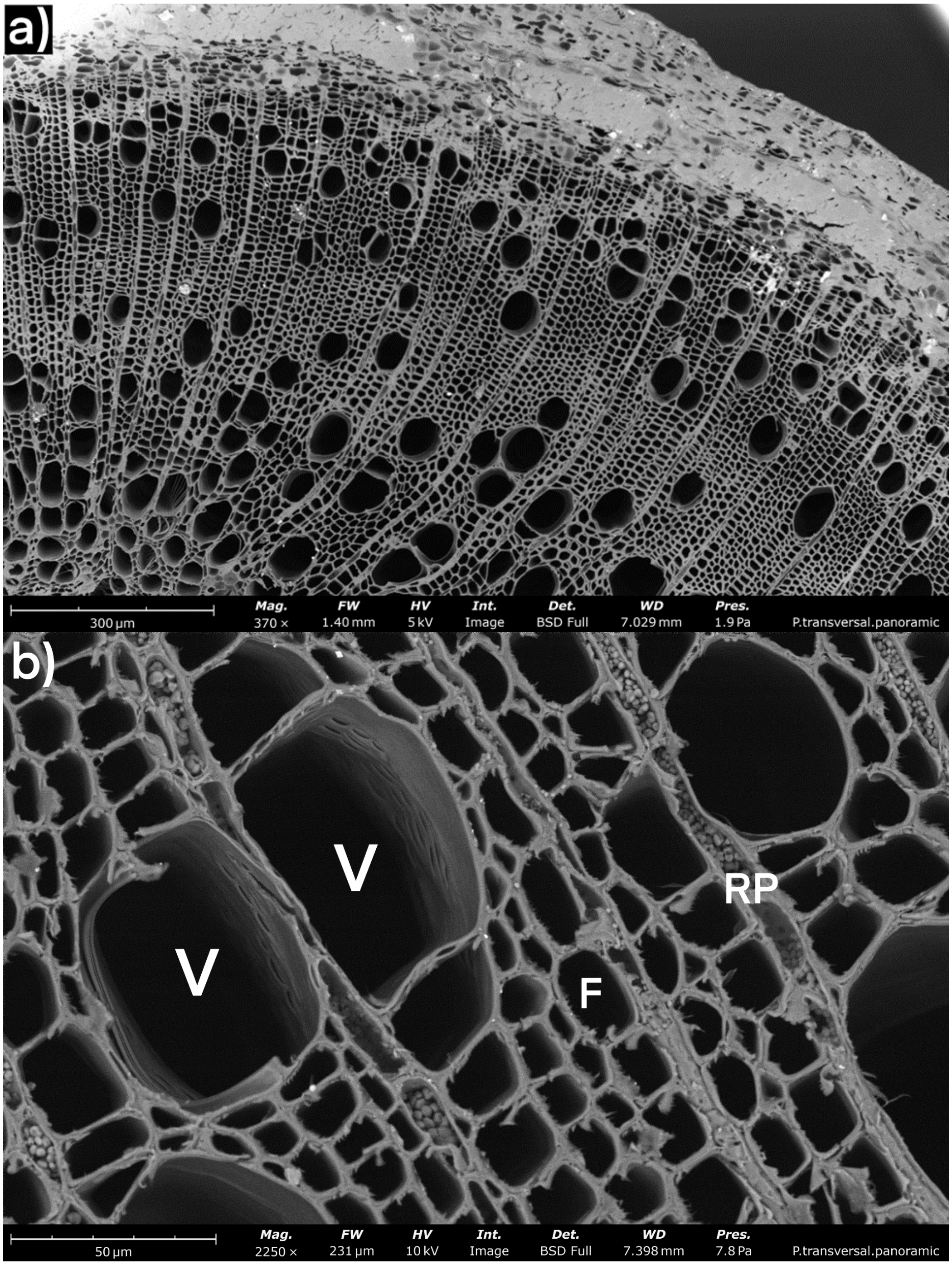

a)
300 µm
Mag. 370 ×
FW 1.40 mm
HV 5 kV
Int. Image
Det. BSD Full
WD 7.029 mm
Pres. 1.9 Pa
P.transversal.panoramic
b)
V
V
F
RP
50 µm
Mag. 2250 ×
FW 231 µm
HV 10 kV
Int. Image
Det. BSD Full
WD 7.398 mm
Pres. 7.8 Pa
P.transversal.panoramic

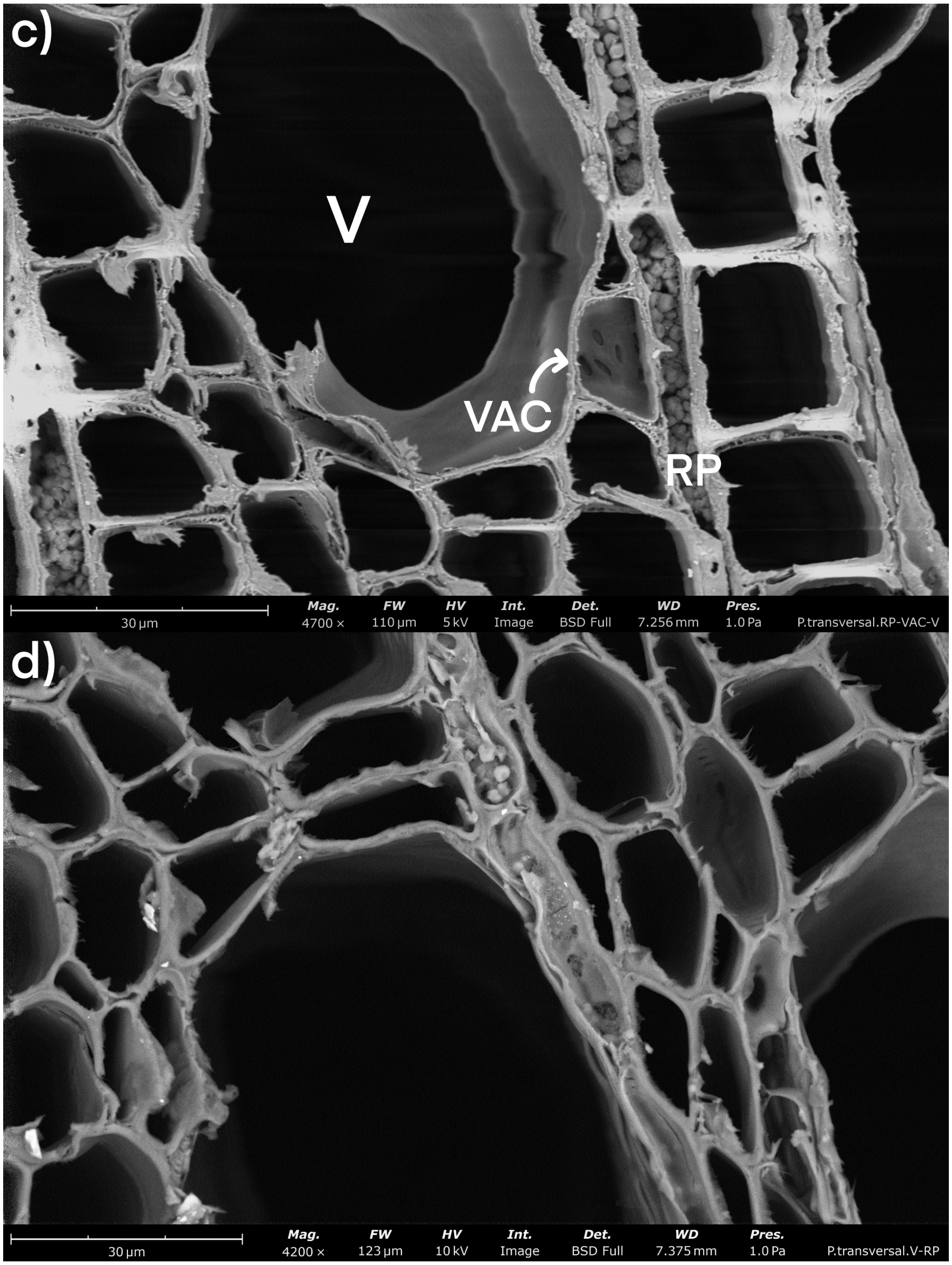

Fig. S3 Additional SEM images.

Scanning Electron Microscopy images of stem cross-section, showing (a) whole cross section and (b-c-d) xylem details. F, fiber; V, vessel; RP, ray parenchyma, VAC, vessel associated cells.

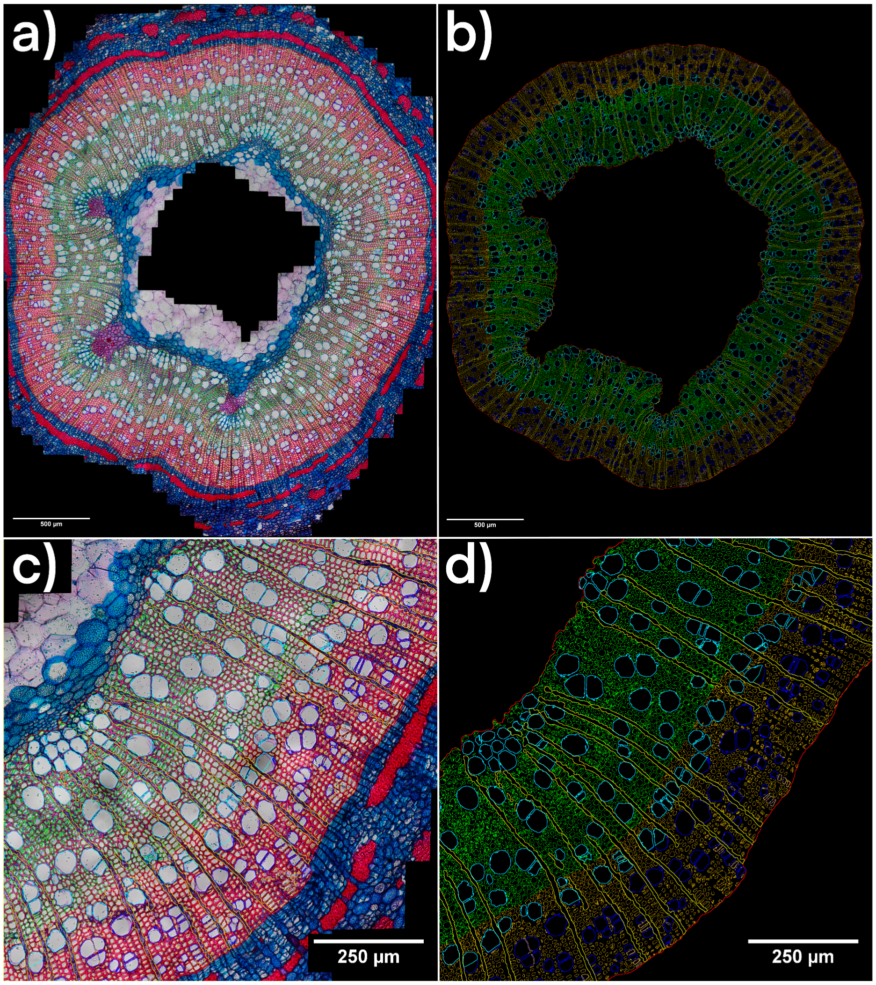

Fig. S4 Ex-vivo anatomy.

Ex-vivo transverse sections of *Populus nigra* stem at approximately 25 cm from the apex. **(a)** Sample double-stained with safranin and astra blue (1:1) represented with overlapped regions of interest (ROIs). **(b)** ROIs of (a) displayed on a black background. **(c)** Details of the sample with overlapped ROIs. **(d)** Details of ROIs of (c) on a black background.

Images were captured on an Olympus BX51 microscope coupled with a canon EOS700D camera. Multiple overlapping captures (n=81 - 1728 x 2304 pixels each) were performed at 200x magnification and later stitched together with no compression (6776 x 8157 pixels) using ptGUI (v8.2.2). Different components were identified through a mix of semi-automated processes (based on color/brightness/size/circularity) and user corrections. The ROIs represent the average distribution of the vast majority of air-filled fibers/embolized vessels (95% threshold) based on the micro CT image analysis, see Fig 4 for more details. ROIs legend: area of embolized vessels in our samples: light blue; area of water-filled vessels in our samples: dark blue; area of air-filled fibers in our samples: green; area of water-filled fibers in our samples: orange; parenchyma: yellow, xylem borders: red.
